# Catalyzing role of erythropoietin on the nitric oxide central pathway during the ventilatory responses to hypoxia

**DOI:** 10.1002/phy2.223

**Published:** 2014-02-14

**Authors:** Nicolas Voituron, Florine Jeton, Yannick Cholley, Raja El Hasnaoui‐Saadani, Dominique Marchant, Patricia Quidu, Fabrice Favret, Jean‐Paul Richalet, Aurélien Pichon

**Affiliations:** 1 Laboratoire “Réponses cellulaires et fonctionnelles à l'hypoxie”, Université Paris 13, Sorbonne Paris Cité, UFR SMBH, EA 2363, Bobigny, 93017, France; 2 Laboratory of Excellence (Labex) GR‐Ex, PRES Sorbonne Paris Cité,; Present Address Laboratoire “Mitochondrie stress oxydant et protection musculaire”, Faculté de Médecine, Université de Strasbourg, EA 3072, Strasbourg, 67085, France

**Keywords:** Erythropoietin, hypoxia, NMDA receptors

## Abstract

The *N*‐Methyl‐d‐Aspartate (NMDA) receptors – neuronal nitric oxide synthase (nNOS) pathway is involved in the ventilatory response to hypoxia. The objective was to assess the possible effect of erythropoietin deficiency and chronic exposure to hypoxia on this pathway during ventilatory response to acute hypoxia. Wild‐type (WT) and erythropoietin‐deficient (Epo‐TAg^h^) male mice were exposed (14 days) either to hypobaric hypoxia (Pb = 435 mmHg) or to normoxia. The ventilation was measured at 21% or 8% O_2_ after injection of vehicle (NaCl), nNOS inhibitor (SMTC) or NMDA receptor antagonist (MK‐801). Nitric oxide production and the expression of NMDA receptor and nNOS were assessed by real‐time RT‐PCR and Western blot analyses in the medulla. At rest, Epo‐TAg^h^ mice displayed normal ventilatory parameters at 21% O_2_ but did not respond to acute hypoxia despite a larger expression of NMDA receptors and nNOS in the medulla. Ventilatory acclimatization to hypoxia was observed in WT but was absent in Epo‐TAg^h^ mice. nNOS inhibition blunted the hypoxic ventilatory acclimatization of WT mice without any effect in Epo‐TAg^h^ mice. Acute hypoxic ventilatory response (HVR) was increased after chronic hypoxia in WT but remained unchanged in Epo‐TAg^h^ mice. Ventilatory response to acute hypoxia was modified by MK‐801 injection in WT and Epo‐TAg^h^ mice. The results confirm that adequate erythropoietin level is necessary to obtain an appropriate HVR and a significant ventilatory acclimatization to hypoxia. Furthermore, erythropoietin plays a potential catalyzing role in the NMDA‐NO central pathway during the ventilatory response and acclimatization to hypoxia.

## Abstract (precis)

e00223

Adequate erythropoietin level is necessary to obtain an appropriate hypoxic ventilatory response and a significant ventilatory acclimatization to hypoxia. Erythropoietin plays a potential catalyzing role on the *N*‐Methyl‐d‐Aspartate (NMDA)‐nNOS central pathway during the ventilatory response and acclimatization to hypoxia.

## Introduction

The acute hypoxic ventilatory response (HVR) is characterized by hyperventilation followed by a relative ventilatory decline (Teppema and Dahan 2010). Then, a time‐dependent increase in ventilation occurs after few hours to several weeks. This ventilatory acclimatization to chronic hypoxia (VAH) is defined by an increase in resting ventilation and in the sensitivity of the respiratory control system (Olson and Dempsey 1978; Bisgard and Neubauer 1995; Prabhakar et al. 1996; Bisgard 2000; Gozal et al. 2000; Powell et al. 2000). This increase in sensitivity includes an enhanced O_2_ peripheral chemosensitivity (Bisgard 2000; Powell 2007) and an increase in central responsiveness to peripheral chemoreceptors input (Powell et al. 2000). Furthermore, the initial increase in HVR contributes to VAH and its magnitude (Bisgard and Neubauer 1995; Powell et al. 1998).

The hypoxia‐inducible factor‐1 (HIF‐1) is the most important protein regulating homeostasis in hypoxia (Gassmann and Soliz 2009). Under chronic hypoxia, HIF‐1 is stabilized and regulates the activation of more than 100 target genes including erythropoietin (Epo) (Yeo et al. 2004; Fandrey et al. 2006; Gassmann and Soliz 2009), the main regulator of red blood cell production, allowing the increase in O_2_ carrying capacity under chronic hypoxia (Gonzalez et al. 1998; Favret et al. 2001; Robach et al. 2004). Epo is also synthesized by the central nervous system (Digicaylioglu et al. 1995). Furthermore, it was suggested that Epo receptors (Epo‐R) are largely distributed in respiratory areas of the brainstem and peripheral carotid bodies (Soliz et al. 2005). While the observation of Soliz and colleagues must be used with caution due to the lack of specificity of the antibody used (Kirkeby et al. 2007), other studies showed that cells in the nervous system express both Epo and its receptor (Masuda et al. 1994; Siren et al. 2001). Therefore, Epo could act on ventilatory control under hypoxic conditions (Soliz et al. 2005) and play a key role for acclimatization to hypoxia in mice (Soliz et al. 2007). Chronic hypoxia leads to an increase in ventilation in wild‐type (WT) mice, which is reduced after neuronal nitric oxide synthase (nNOS) inhibition (El Hasnaoui‐Saadani et al. 2007). Moreover, it has been proposed that activation of *N*‐Methyl‐d‐Aspartate receptors (NMDA‐R) could be involved in the early HVR (Gozal et al. 2000). When glutamate binds to NMDA‐R, the intracellular calcium concentration increases, which may activate nNOS and induce a rise in nitric oxide (NO) production, then act as an excitatory neurotransmitter in HVR (Ogawa et al. 1995). Therefore, chronic hypoxia was shown to stimulate the central pathway including NMDA‐R, nNOS, and NO production. In addition, Epo was shown to increase NO production in rat hippocampus by activating voltage‐gated Ca^2+^ channels, but not through NMDA receptors (Yamamoto et al. 2004).

Considering the effects of the NMDA‐R‐nNOS pathway on VAH in WT mice (El Hasnaoui‐Saadani et al. 2007) and the possible link between Epo and NO production, we aimed to investigate the role of Epo and its interaction with the central NMDA‐NO central pathway on HVR in mice exposed to either chronic normoxic (NX‐exposed) or hypoxic (HX‐exposed) conditions. We supposed that Epo could play a key role in VAH and HVR mechanisms by increasing ventilatory responses to hypoxia through the central NMDA‐NO pathway. To test our hypothesis, we measured ventilatory parameters in normoxia and acute hypoxia after injection of vehicle, NMDA‐R antagonist (MK‐801) and nNOS inhibitor (SMTC) in WT and Epo‐deficient (Epo‐TAg^h^) NX‐exposed or HX‐exposed mice.

## Methods

### Ethics statement

Experimental protocols were approved by The Ethics Committee in Animal Experiment Charles Darwin (Ce5/2011/05), done in accordance with the European Communities Council Directive of September 22, 2010 (2010/63/EU) for animal care, and conducted in accordance with French laws for animal care (authorization number: A‐93‐086 for NV and A‐93‐072 for AP).

### Animals

All experiments were performed in WT (*n* = 64) and Epo‐TAg^h^ (*n* = 52) male adult littermates (≈10 weeks at the beginning of the study) born from Bl6/CBA strain. The Epo‐TAg^h^ mice present a targeted disruption in the 5′ untranslated region of the Epo gene that reduces the whole‐body Epo expression (Binley et al. 2002). Mean body weight and hematocrit was 25.2 ± 0.5 g and 41.2 ± 4.4% for WT and 28.0 ± 0.8 g and 20.7 ± 2.2% for Epo‐TAg^h^ mice, respectively.

Mice were exposed to either chronic normoxic (NX‐exposed) or hypoxic (HX‐exposed) conditions. The HX‐exposed group was housed for 14 days in a hypobaric chamber maintained at a pressure of 435 mmHg (≈4460 m) by a vacuum source at flow rates sufficient to prevent CO_2_ buildup. The chamber was returned to sea level (760 mmHg) twice a week for 30 min to clean the cages and to renew the water and food. The NX‐exposed group was kept outside of the hypobaric chamber at 760 mmHg. All animals were housed in a 12 h/12 h light/dark cycles and had ad libitum access to water and food.

Ninety‐two mice were used to analyze ventilatory parameters and 24 mice were used to quantify NMDA‐R protein and mRNA and nitrate‐nitrite (NOx) as markers of nitric oxide (NO) production.

### Ventilatory parameters

**Figure 1.**
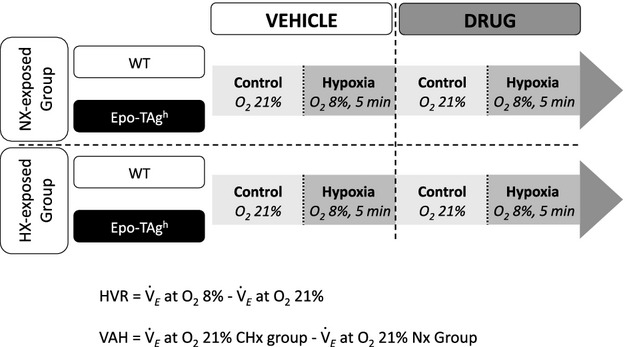
Study design. The ventilatory parameters were evaluated by plethysmographic approach in non‐anesthetized and unrestrained mice exposed to either chronic normoxic (NX‐exposed) or hypoxic (HX‐exposed) conditions. The acute ventilatory response to hypoxia was checked by replacing air (control) by hypoxic gas mixture (hypoxia, O_2_ 8 %, 5 min) before (vehicle) and after drug injection.

In non‐anesthetized and unrestrained mice, breathing parameters were recorded by whole‐body plethysmography as previously described (Bartlett and Tenney 1970; Menuet et al. 2011). Briefly, the plethysmographic chamber (200 mL) was connected to a differential pressure transducer (model DP 45‐18; Validyne Engineering Northridge, CA), which measured pressure fluctuations within the closed chamber relative to a reference chamber of the same volume. Breathing parameters were recorded by a BIOPAC data analysis system (BIOPAC System Inc., Santa Barbara, CA). Two or three days before plethysmographic recordings for the NX‐exposed groups or before entering the hypobaric chamber for HX‐exposed groups, mice were accustomed to stay in the plethysmograph chamber to reduce stress effect on breathing. To check the acute ventilatory responses to hypoxia (Fig. 1), air was replaced by hypoxic gas mixture (O_2_ 8%, 5 min). Routinely, WT and Epo‐TAg^h^ mice were alternatively recorded and subjected to the same environmental challenges. Only periods of breathing without body movements were analyzed. We measured respiratory frequency (*f*_R_, in cycle per min, c·min^−1^), tidal volume (V_T_, *μ*L) normalized as the ratio V_T_ divided by body weight (V_T_, *μ*L·g^−1^), and minute ventilation ((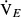, mL·g^−1^·min^−1^). HVR was calculated as the difference in 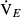 between 8% and 21% O_2_. VAH was calculated as the difference in 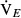 in normoxic conditions (21% O_2_) between NX‐exposed and HX‐exposed groups.

### Drugs

In order to test the role of medullary NO production, mice were subjected to a single intraperitoneal (i.p.) injection of either vehicle (NaCl; *n* = 10 for WT NX‐exposed and for Wt HX‐exposed; *n* = 8 for Epo‐TAg^h^ NX‐exposed mice and for Epo‐TAg^h^ HX‐exposed mice), MK‐801 (Sigma Aldrich, St. Louis, MO, NMDA‐R antagonist, 3 mg·kg^−1^; *n* = 8 for WT NX‐exposed and for Wt HX‐exposed; *n* = 6 for Epo‐TAg^h^ NX‐exposed mice and for Epo‐TAg^h^ HX‐exposed mice) or SMTC (Sigma Aldrich, nNOS inhibitor, 10 mg·kg^−1^; *n* = 9 for WT NX‐exposed and *n* = 7 for Wt HX‐exposed; *n* = 6 for Epo‐TAg^h^ NX‐exposed mice and for Epo‐TAg^h^ HX‐exposed mice). Breathing records were performed during control period and during the last minute of the acute hypoxic exposure. During the control period, breathing parameters were recorded three times at 10 min intervals and averaged. A first set of measurement was performed prior drug injection and a second one was performed 30 min (SMTC) or 1 h (MK801) after injection.

### Biochemical analysis

As previously reported (El Hasnaoui‐Saadani et al. 2007, 2009), at the end of either chronic normoxic or hypoxic exposure, mice were killed by cervical elongation and then beheaded. The brain was rapidly removed and the medulla quickly frozen in liquid nitrogen and stored at −80°C until use.

#### Medulla RNA Extraction and Real‐Time RT‐PCR

Total RNA was isolated from the medulla using TRIZOL Reagent (Invitrogen, Carlsbad, CA) and digested with RNase free DNase I (Invitrogen) for 1 h 30 min at room temperature to remove any contaminating genomic DNA. cDNA synthesis was performed using the SuperScript III First‐Strand Synthesis System for RT‐PCR (Invitrogen) according to the manufacturer's instructions (5 *μ*g total RNA/20 *μ*L cDNA synthesis reactions, 50 *μ*mol/L oligo(dT)20, 10 mmol/L dNTP mix).

Then, duplicate real‐time PCR was performed on generated cDNA using the Light Cycler FastStart DNA Master SYBR Green I (Roche Biochemicals, Stockholm, Sweden) and gene‐specific PCR primers (El Hasnaoui‐Saadani et al. 2007) for quantitative analyses of genes of interest. PCR amplifications were performed according to the manufacturer's instructions. The results were represented as threshold cycle numbers (Ct values). Control cDNA of normoxic WT mice medulla was used to create standard curves. Relative amounts of mRNA, normalized by *β*‐actin were calculated from Ct values, according to the manufacturer's description (Roche Biochemicals).

#### Medulla Protein Extraction and Western Blot Analysis

Twenty‐four medulla samples of WT and Epo‐TAg^h^ mice NX‐exposed or HX‐exposed (six mice for each group) were homogenized in ice‐cold buffer supplemented with a protease inhibitor cocktail (Sigma Aldrich): (a) 20 mmol/L 4‐(2‐hydroxyethyl)‐1‐piperazineethanesulfonic acid, 1.5 mmol/L MgCl2, 0.2 mmol/L ethylenediaminetetraacetic acid, 0.1 mmol/L NaCl, 0.2 mmol/L dithiothreitol, 0.5 mmol/L NaVO4, pH 7.5 for NMDA‐R measurement, (b) 50 mmol/L Tris HCl, 150 mmol/L NaCl, 1% Triton X‐100, 0.1% sodium deoxycholate, pH 7.2 for nNOS measurements. Homogenates were centrifuged at 10.000*g* for 30 min at 4°C. Then, supernatants were collected and stored at −20°C until use.

Protein of each studied sample (100 *μ*g) was separated by electrophoresis on 7.5% (nNOS) or 10% (NMDA‐R) sodium dodecyl sulphate polyacrylamide gel and transferred to polyvinylidene difluoride (PVDF) membrane (Millipore SAS, Molsheim, France). In addition, positive controls were also loaded, and precision prestained standards were used as molecular weight markers (Bio‐Rad Laboratories, Marnes‐la‐Coquette, France). Membranes were kept overnight in 5% BSA (bovine serum albumin) / TBS (tris‐buffered saline)‐0.5% Tween 20 at 4°C to block nonspecific binding. Membranes were then incubated overnight at 4°C with each primary antibody (nNOS [sc‐648]; NMDA‐R [sc‐9058]; Santa Cruz Biotechnology, Santa Cruz, CA) diluted at 1/500 in 1% BSA/TBS‐0.5% Tween 20 (TBS‐T). Membranes were washed with TBS‐T and incubated for 2 h at room temperature with either 1:500 anti‐rabbit or 1:500 anti‐mouse IgG antibody‐horseradish peroxidase conjugate depending on the species of the primary antibodies (Santa Cruz Biotechnology). Immunodetection was accomplished using ECL Western blot analysis detection kit (Santa Cruz Biotechnology). Membranes were then probed with anti *β*‐actin (Santa Cruz Biotechnology) as internal control. Densitometric scanning and image analysis, using ImageJ (National Institute of Health, Bethesda, MD), were done to quantify the specific protein expression of each sample compared to internal control. Data were then expressed as the ratio of the protein of interest density to that of the *β*‐actin.

#### NOx Colorimetric Assay

The total amount of NO in the medulla samples (*n* = 6 for WT and *n* = 6 for Epo‐TAg^h^) was assessed by the Griess reaction using a colorimetric assay kit (nitrate/nitrite colorimetric assay kit; Cayman Chemical, MI) that detects nitrite (NO_2_^−^) and nitrate (NO_3_^−^), which are stable reaction products of NO. Because the relative proportion of each metabolite may vary, the index of total NO production is best assessed by their sum (NOx). Hence, homogenates and assay procedure were carried out following the manufacturer's instructions. Briefly, cerebral cortex samples were homogenized in four volumes (W/V) of phosphate buffered saline (pH 7.6) at 4°C, ultracentrifuged at 100.000*g* for 60 min at 4°C. NOx determination was carried out in supernatants. The nitrate reductase reaction was first employed to convert nitrate to nitrite followed by Griess reaction in order to measure metabolites by photometric absorbance using an Enzyme‐Linked Immunosorbent Assay plate reader. The optical density was measured at 540 nm. The quantity of NOx detected in each sample was compared to a nitrite standard curve. Finally, data were expressed as the ratio of the quantity of NOx to that of total protein.

### Statistical analysis

Values are presented as mean ± standard deviation (SD). Kolmogorov–Smirnov test assessed the normality of distribution. The effects of acute hypoxia (F_I_O_2_ 8% vs. 21%) on ventilatory and biochemical parameters in WT and Epo‐TAg^h^ mice were evaluated (i) after exposure to either chronic normoxic or hypoxic conditions and/or (ii) drugs injection (NaCl, SMTC or MK‐801, Fig. 1). Differences were tested by a multivariate analysis of variance (MANOVA) with Greenhouse and Geisser adjustments. Newman–Keuls post hoc tests were used to assess specific differences between groups. All analyses were performed with the Statistica software (Stat Soft, Tulsa). Differences were considered significant when *P* < 0.05.

## Results

### Effect of Epo deficiency and exposure to chronic hypoxia on normoxic ventilatory parameters

At baseline, ventilatory parameters were similar in WT and Epo‐TAg^h^ mice (Table 1) and rectal temperatures were not different (35.1 ± 0.2°C and 35.2 ± 0.9°C, respectively). After 14 days in the hypobaric chamber, 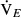 of WT mice was significantly higher (VAH) as compared to NX‐exposed mice. This increase in 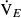 was mediated exclusively by an increase in V_T_, as breathing frequency was unaltered in HX‐exposed mice. In contrast, ventilatory parameters were not altered in Epo‐Tag^h^ HX‐exposed mice. Rectal temperatures of WT and Epo‐TAg^h^ mice were not modified after chronic hypoxia (35.7 ± 0.6°C and 35.7 ± 0.7°C, respectively) as compared to mice housed in normoxia.

**Table 1.** Ventilatory parameters in wild‐type and Epo‐TAg^h^ mice exposed either to normoxic (NX‐exposed) or hypoxic (HX‐exposed) conditions tested in normoxia (F_I_O_2_ = 21%) and acute hypoxia (F_I_O_2_ = 8%).

	Acclimatization	F_I_O_2_ (%)	Wild type mice			Epo‐TAg^h^ mice		
			NaCI	SMTC	MK‐801	NaCI	8M1C	MK‐801
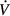 _E_ (mL·g^−1^·min^−1^	NX‐exposed	21	2.26 ± 0.48	2.20 ± 0.57	2.90 ± 0.38	2.17 ± 0.53	2.15 ± 0.53	2.72 ± 0.16
		8	3.61 ± 1.14*	3.46 ± 0.60*	2.03 ± 0.50*	2.59 ± 0.44	3.04 ± 0.27	3.04 ± 0.45*
	HX‐exposed	21	3.51 ± 0.77*	2.87 ± 0.81	3.16 ± 0.83	2.57 ± 0.37	2.45 ± 0.36	3.37 ± 0.29
		8	7.27 ± 1.07*^,^*	6.31 ± 0.74*^,^*^,^*	3.15 ± 0.66*^,^*	3.86 ± 0.52*^,^*	3.60 ± 0.40*	3.72 ± 1.14
V_T_ (*μ*L·g^−1^)	NX‐exposed	21	8.63 ± 1.26	7.20 ± 1.43	10.97 ± 1.15*	7.62 ± 1.09	7.61 ± 1.34	12.32 ± 0.97*
		8	11.28 ± 2.37*	12.19 ± 1.83*	9.12 ± 1.37	7.68 ± 1.28	9.03 ± 0.57	9.08 ± 2.06*
	HX‐exposed	21	11.96 ± 1.24*	9.55 ± 1.66*^,^*	14.27 ± 3.08*^,^*	9.13 ± 1.97	7.95 ± 0.68	17.32 ± 2.61*^,^*
		8	18.05 ± 2.38*^,^*	15.76 ± 1.26*^,^*^,^*	11.38 ± 2.04*^,^*	10.46 ± 1.55*	9.25 ± 0.80	16.14 ± 4.20*^,^*
f_R_ (c·min^−1^)	NX‐exposed	21	261 ± 34	307 ± 53	268 ± 47	284 ± 54	282 ± 47	221 ± 19
		8	318 ± 54	284 ± 28	226 ± 56*	338 ± 34	337 ±18	244 ± 27*
	HX‐exposed	21	292 ± 49	293 ± 55	222 ± 34*	289 ± 48	309 ± 42	198 ± 34*
		8	404 ± 21^1,^*	407 ± 20*^,^*	280 ± 49*	370 ± 11^1^	389 ± 26*	227 ± 34*

Values are mean ± SD of ventilation (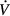
_E_), tidal volume (V_t_) and respiratory frequency (*f*_R_). Significant differences are indicated as follows.

* *P *< 0.05 versus 21% for the same group.

* *P* < 0.05 effect of exposure to chronic hypoxia in the same strain and the same FIO_2_.

* *P* < 0.05 versus NaCI in the same strain for the same FIO_2_.

### Effect of exposure to chronic hypoxia on HVR

**Figure 2.**
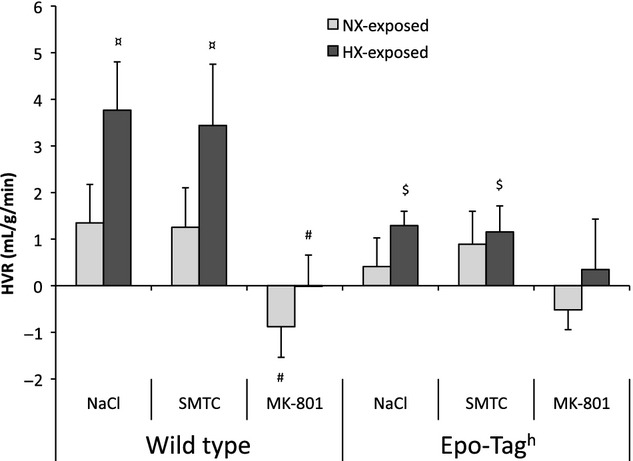
Effect of exposure to chronic hypoxia and drugs injections on HVR. Hypoxic ventilatory response was the difference between the recorded value at 8% O_2_ and 21% O_2_. Given values are obtained before and after drugs injections (NaCl, SMTC or MK‐801). Values are means ± SD. ^¤^*P* < 0.05 effect of exposure to chronic hypoxia in the same strain. ^#^*P* < 0.05 versus NaCl in the same strain. ^$^*P* < 0.05 versus wild‐type (WT) animals.

In WT NX‐exposed mice, exposure to acute hypoxia (8% O_2_) led to an increase in V_T_ and 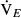 (HVR) whereas Epo‐TAg^h^ mice did not respond (Table 1, Fig. 2). HX‐exposed WT mice showed a larger HVR than without acclimatization (Table 1, Fig. 2). In Epo‐TAg^h^ HX‐exposed mice, acute hypoxia (8% O_2_) led to an increase in *f*_R_ and 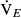 (Table 1).

### Effect of injection of nNOS inhibitor and NMDA receptor antagonist on normoxia‐exposed mice

In WT and Epo‐TAg^h^ mice housed in normoxic conditions, injection of SMTC had no effect on ventilatory parameters (Table 1) whereas V_T_ was significantly increased after MK‐801 injection in both mice strains. During the acute hypoxic challenge, SMTC had no effect on HVR (Table 1, Fig. 2), while it was completely abolished by MK‐801 in WT NX‐exposed mice.

### Effect of interaction between drug injection and exposure to chronic hypoxia

#### Effect of SMTC after exposure to chronic hypoxia

**Figure 3.**
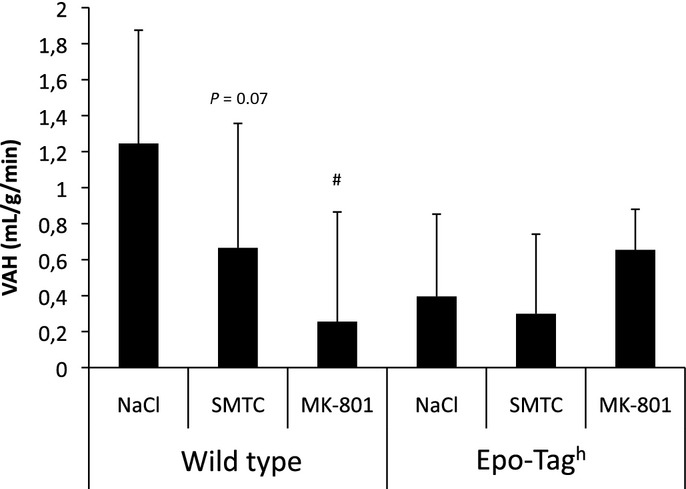
Effect of chronic hypoxia and drug injections on VAH. Difference in minute ventilation at 21% O_2_ between mice housed in normoxia and mice exposed to chronic hypoxia in wild‐type (WT) and Epo‐TAg^h^ mice, representing the ventilatory acclimatization to chronic hypoxia (VAH) and after drug injections (NaCl, SMTC or MK‐801). ^#^*P* < 0.05 versus NaCl in the same strain. Values are means ± SD.

SMTC injection reduced the increase in 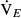 induced by 14 days of acclimatization to chronic hypoxia in WT mice (Table 1) and tended to decrease the VAH (*P* = 0.07, Fig. 3). However, HVR persisted without change after nNOS inhibition (Fig. 2) despite a slight reduction in V_T_ and 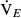 as compared to vehicle in WT mice HX‐exposed during acute response to hypoxia (F_I_O_2_ = 8%, Table 1). No effect of SMTC was reported on VAH in HX‐exposed Epo‐TAg^h^ mice after 14 days of chronic hypoxia (Table 1, Fig. 3).

#### Effect of MK‐801 after exposure to chronic hypoxia

MK‐801 injection reduced the VAH induced by exposure to chronic hypoxia in WT mice (Table 1, Fig. 3). The injection of MK‐801 induced a significant decrease in *f*_R_ and an increase in V_T_ as compared to vehicle in HX‐exposed WT and Epo‐TAg^h^ mice at 21% O_2_ (Table 1). During acute hypoxia (8% O_2_), MK‐801 injection led to a significant decrease in 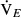, V_T_ and *f*_R_ in HX‐exposed WT mice as compared to vehicle (Table 1). In HX‐exposed Epo‐TAg^h^ mice, 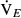 was unchanged as compared to vehicle despite an increase in V_T_ and a decrease in *f*_R_ (Table 1). HVR after exposure to chronic hypoxia was totally abolished after MK‐801 injection as compared to vehicle in WT mice (Fig. 2).

### NMDA‐R and nNOS expression in medulla

At baseline, in the medulla of mice housed in normoxia, NMDA‐R and nNOS mRNA and protein expressions (Table 2) were higher in Epo‐TAg^h^ mice than in WT mice. After exposure to chronic hypoxia, mRNA and protein levels of NMDA‐R were significantly higher in WT mice as compared to mice housed in normoxia, while it remained unchanged in Epo‐TAg^h^ mice. The nNOS mRNA and protein levels were higher in WT whereas only nNOS protein was higher in Epo‐TAg^h^ mice exposed to chronic hypoxia compared with those housed in normoxia (Table 2).

**Table 2.** Expression of mRNA and protein of NMDA‐R1 and nNOS in the medulla and NO production in WT and Epo‐TAg^h^ mice exposed either to normoxic (NX‐exposed) or hypoxic (HX‐exposed) conditions.

	Wild‐type		Epo‐TAg^h^	
	NX‐exposed	HX‐exposed	NX‐exposed	HX‐exposed
NMDA‐R1 mRNA/*β*‐actin	8.26 ± 1.39	20.81 ± 1.97*	26.62 ± 0.93*	17.67 ± 2.21
NMDA‐R1 protein/*β*‐actin	2.94 ± 0.16	4.14 ± 0.60*	4.09 ± 0.19*	4.16 ± 0.99
nNOS mRNA/*β*‐actin	0.96 ± 0.19	1.72 ± 0.23*	2.30 ± 0.21*	2.25 ± 0.05
nNOS protein/*β*‐actin	0.57 ± 0.07	1.71 ± 0.11^1^	1.03 ± 0.12^2^	2.01 ± 0.36*
NOx (*μ*mol·*μ*L^−^*	103 ± 6	146 ± 4*	85 ± 6	242 ± 20*^,^*

Values are Mean ± SD of the expression of mRNA and protein of NMDA‐R1 and nNOS and production of NO (NOx, sum of NO_2_^−^, and NO_3_^−^). Significant differences are indicated as follows.

* *P* < 0.05 effect of exposure to chronic hypoxia in the same strain.

* *P* < 0.05 versus wild‐type strain with the same exposition (NX or HX).

### NO production

Concentrations of NO metabolites were the same between WT and Epo‐TAg^h^ mice housed in normoxia (Table 2). After 14 days of hypobaric hypoxia, NO metabolites were higher than in normoxia in both strains of mice and were significantly larger in Epo‐TAg^h^ mice than in WT mice (Table 2).

## Discussion

This study was designed to evaluate the interaction between Epo and NMDA‐NO central pathway in ventilatory response to acute hypoxia and the modulation of this phenomenon by prior exposure to chronic hypoxia. Our results confirm that adequate Epo level is necessary to obtain normal HVR and VAH. Moreover, our results show that MK‐801 blocks HVR and VAH and that SMTC have only an effect on VAH, suggesting that the effect of NMDA and NO pathways might be independent.

### Methodological considerations

Experiments were performed on Epo‐deficient SV‐40 T antigen mice that display a whole‐body reduced Epo expression (Binley et al. 2002). The Epo‐deficient mice display also a very low hematocrit (around 20%) and did not develop polycythemia after exposure to chronic hypoxia contrary to WT mice (Macarlupu et al. 2006b). Indeed, hemoglobin concentration as well as hematocrit did not change significantly after 14 days of chronic hypoxia compared to normoxic values (Macarlupu et al. 2006b). Therefore, we cannot exclude that the observed effects are due to chronic anemia or the combined effect of Epo deficiency and anemia.

It is known that carotid bodies contribute to Epo regulation of ventilation (Soliz et al. 2005). As the pharmacological substances used in this study were injected intraperitoneally we cannot exclude a systemic effect, which could not only be limited to the medulla but could also affect peripheral chemoreceptors or other areas of the central nervous system. Consequently, we cannot determine which tissues are responsible for the difference observed in the ventilatory response of Epo‐TAg^h^ mice (medulla and/or carotid bodies).

### Effect of Epo deficiency under normoxic conditions

Epo deficiency did not modify breathing parameters and central temperature in resting condition (21% O_2_) at least in male mice. In Epo‐deficient mice, no significant HVR was found. These results confirm that Epo is necessary to obtain a correct HVR. Indeed, it has been previously proposed that Epo exerts a key role in HVR (Soliz et al. 2005) and improves this response in female mice (Soliz et al. 2009).

### Effect of exposure to chronic hypoxia on NO pathway in WT and Epo‐deficient mice

In WT mice, exposure to chronic hypoxia leads to an increase in NMDA‐R expression and NO production in the medulla. These results suggest that the intracellular signaling pathways that are used by Epo at the nervous system level are similar to those implicated in tissue protection. Indeed, Epo modulates the activity of calcium channels through phospholipase C, thereby reducing the release of excitatory neurotransmitters and increasing NO production (Brines and Cerami 2005). The decrease in the excitatory neurotransmitters could be at the origin of the observed increase in NMDA‐R expression in WT mice, in order to increase the effectiveness of the neurotransmitter. In Epo‐TAg^h^ mice, the absence of modifications in NMDA‐R expression after exposure to chronic hypoxia suggests that sufficient level of Epo is necessary to maintain a correct release of neurotransmitters. Indeed, Epo directly influences neurotransmitter release as well as neuronal activity (Koshimura et al. 1999; Kawakami et al. 2000). The increase in NO production in Epo‐deficient mice could suggest that other pathways could be involved.

### Effect of exposure to chronic hypoxia on VAH and HVR in WT and Epo‐deficient mice

As already shown by our team (El Hasnaoui‐Saadani et al. 2007) and others (Powell et al. 1998), acclimatization to chronic hypoxia leads to: (i) a significant increase in ventilation (VAH) mainly due to an increase in V_T_ (Table 1) and (ii) a significant increase in HVR (Fig. 3). In WT mice, plasma Epo is known to increase HVR by stimulating peripheral chemoreceptors (Soliz et al. 2005). In Epo‐TAg^h^ mice, chronic hypoxia did not induce a significant VAH despite unchanged NMDA‐R but increased nNOS expression and NOx production in the medulla (Table 2). Nevertheless, Epo‐TAg^h^ mice displayed a slight but significant increase in V_T_ and 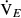 at 8% O_2_ after chronic hypoxia exposure. These results suggest a potential role of nNOS and NOx in HVR after exposure to chronic hypoxia in Epo‐TAg^h^ mice that partially compensates the lack of high Epo concentration. Another explanation could be that 8% O_2_ was too severe for these transgenic mice to increase ventilation and that the related energetic cost of breathing was too high, given their low oxygen‐carrying capacity. Exposure to chronic hypoxia did not affect rectal temperature neither in WT nor in Epo‐TAg^h^ mice. Therefore, metabolic rate seems unaffected by chronic hypoxia and/or Epo deficiency.

### Effect of drug injection on VAH and HVR in WT mice

#### Effects of nNOS on ventilatory control in hypoxia

The present results confirm previous studies from our group (El Hasnaoui‐Saadani et al. 2007) and support the idea that the increase in nNOS expression leading to a rise in NO modulates the ventilatory pattern mainly through an increase in Vt and contributes to VAH without any effect on HVR in nonacclimatized WT mice (for more details see El Hasnaoui‐Saadani et al. (2007)).

#### Effects of NMDA‐R on ventilatory control in hypoxia

It was clearly demonstrated that in the first stages of hypoxia, peripheral chemoreceptor stimulation elicits a rapid ventilatory increase by enhancing glutamate release (Mizusawa et al. 1994) and activation of postsynaptic NMDA‐R in the nucleus of the solitary tract (Ohtake et al. 1998). Indeed, Ohtake et al. (1998) have shown that NMDA‐R protein level increased after hypoxic exposure in the nucleus of the solitary tract of adult rats, which is in agreement with a role of NMDA in HVR in mammals (Gozal et al. 2000). These results were confirmed by our observation in WT mice with or without exposure to chronic hypoxia. Reid and Powell (2005) also demonstrated that systemic NMDA‐R blockade (MK801) eliminated HVR in normoxic and chronically hypoxic rats.

The rise in NMDA‐R in chronically hypoxic mice could explain the larger response to NMDA‐R blockade on ventilatory parameters after acclimatization. Taken together, these results suggest that NMDA‐R are involved in HVR after chronic hypoxia and support the hypothesis of NMDA‐R plasticity with acclimatization to hypoxia. Moreover, the activation of NMDA‐R, enhancing HVR, seems also to be linked to nNOS expression but only after exposure to chronic hypoxia. Indeed, a small but significant decrease in ventilation was observed after SMTC injection in acclimatized WT mice exposed to acute hypoxia, suggesting that nNOS was partially involved in HVR after acclimatization. Therefore, our results suggest that, in chronically hypoxic mice, the increase in NMDA‐R may induce a rise in intracellular calcium leading to calmodulin release, which could reinforce the role of nNOS in VAH after acclimatization to hypoxia. Surprisingly, MK‐801 induced a significant increase in V_T_ in WT mice before and after acclimatization in normoxic conditions suggesting that compensatory mechanisms overwhelmed NMDA‐R inefficiency.

### Effect of drug injection on VAH and HVR in Epo‐deficient mice

#### Role of nNOS and NOx on ventilatory control in hypoxia

In Epo‐TAg^h^ mice housed at sea level, we observed that SMTC injection had no effect on ventilatory parameters (Table 1) despite a larger expression of nNOS in the medulla as compared to WT mice. Ward et al. (2005) showed that hypoxia induced a significant increase in nNOS protein levels (~10‐fold) whereas NOS activity increased less than 1.5‐fold suggesting a difference between the expression of the protein and its activity. Our results suggest that the activity of nNOS in the medulla of Epo‐TAg^h^ mice could be lower than in WT mice, probably due to Epo deficiency, and, therefore, lead to a lower HVR. However, the slight but significant increase in V_T_ and 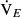 at 8% O_2_ observed after exposure to chronic hypoxia in Epo‐TAg^h^ mice disappeared after SMTC injection (Table 1). As observed in WT mice (but with lower amplitude) it seems, therefore, that nNOS and NOx could be partially involved in HVR after chronic hypoxia in Epo‐TAg^h^ mice.

#### Role of NMDA‐R on ventilatory control in hypoxia

The injection of MK‐801 had no effect on 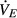 in Epo‐TAg^h^ mice. However, significant and opposite changes were observed on V_T_ and *f*_R_ suggesting that NMDA‐R are involved in the regulation of the ventilatory pattern in these Epo‐deficient mice. Indeed, MK‐801 greatly decreased *f*_R_ with a concomitant increase in V_T_ at 21 and 8% O_2_ before and after exposure to chronic hypoxia (Table 1). This effect of MK‐801 in Epo‐TAg^h^ mice was the same, in a larger extent, as the one observed in WT mice and reinforces the hypothesis of a potential compensatory mechanism of NMDA‐R blockade to maintain baseline ventilation before and furthermore after chronic hypoxia.

In Epo‐TAg^h^ mice, NMDA‐R antagonist had no significant effect on ventilation when compared to vehicle despite a significant difference after SMTC injection (Fig. 3). This result suggests that NMDA‐R seems to be also partially involved in HVR in Epo‐TAg^h^ mice.

## Conclusion

This study provides evidence that nNOS is partially involved in VAH in Epo‐TAg^h^ mice. NMDA receptors are increased in the medulla of Epo‐TAg^h^ mice exposed to chronic hypoxia and could also be involved in VAH. Moreover, NMDA receptors are crucial for HVR. These results are, therefore, consistent with the hypothesis of central nervous system plasticity not only partially involving nNOS for VAH and requiring NMDA receptors for HVR and VAH, but also with a potential important catalyzing role of Epo.

## Translational perspective

The erythropoietic system is known to stimulate production of red blood cells in hypoxic conditions. Besides this well‐known role, Epo synthesised by neurons and astrocytes modulates the ventilatory response to hypoxia. This effect has been attributed to the presence of Epo receptors in both brainstem respiratory centres and carotid bodies but little is known regarding the mechanisms involved in hypoxic ventilatory response and ventilatory acclimatisation to hypoxia. Using Epo‐deficient mice, we showed that Epo could play a key‐regulating role in the neural control of ventilatory acclimatisation to hypoxia and hypoxic ventilatory response probably via a catalysing role on the nitric oxide central pathway. These novel findings are relevant to better understand the respiratory regulations, especially those occurring during Epo deficiency.

## Acknowledgments

Authors thank Sonia and Samira Varela for the animal's care and Rosa Cardenas Alayza for technical help and support.

## Conflict of Interest

None declared.
